# The association between disease activity and NT-proBNP in 238 patients with rheumatoid arthritis: a 10-year longitudinal study

**DOI:** 10.1186/ar2442

**Published:** 2008-06-23

**Authors:** Sella A Provan, Kristin Angel, Sigrid Ødegård, Petter Mowinckel, Dan Atar, Tore K Kvien

**Affiliations:** 1 Department of Rheumatology, Diakonhjemmet Hospital, Box 23 Vindern, N-0319 Oslo, Norway; 2 Faculty of Medicine, University of Oslo, Pb1018 0316 Oslo, Norway; 3 Aker University Hospital, Division of Cardiology, University of Oslo, Trondheimsveien 235, 0514 Oslo, Norway

## Abstract

**Introduction:**

Disease activity in patients with rheumatoid arthritis (RA) is associated with increased cardiovascular morbidity and mortality, of which N-terminal pro-brain natriuretic peptide (NT-proBNP) is a predictor. Our objective was to examine the cross-sectional and longitudinal associations between markers of inflammation, measures of RA disease activity, medication used in the treatment of RA, and NT-proBNP levels (dependent variable).

**Methods:**

Two hundred thirty-eight patients with RA of less than 4 years in duration were followed longitudinally with three comprehensive assessments of clinical and radiographic data over a 10-year period. Serum samples were frozen and later batch-analyzed for NT-proBNP levels and other biomarkers. Bivariate, multivariate, and repeated analyses were performed.

**Results:**

C-reactive protein (CRP) levels at baseline were cross-sectionally associated with NT-proBNP levels after adjustment for age and gender (*r*^2 ^adjusted = 0.23; *P *< 0.05). At the 10-year follow-up, risk factors for cardiovascular disease were recorded. Duration of RA and CRP levels were independently associated with NT-proBNP in the final model that was adjusted for gender, age, and creatinine levels (*r*^2 ^adjusted = 0.38; *P *< 0.001). In the longitudinal analyses, which adjusted for age, gender, and time of follow-up, we found that repeated measures of CRP predicted NT-proBNP levels (*P *< 0.001).

**Conclusion:**

CRP levels are linearly associated with levels of NT-proBNP in cross-sectional and longitudinal analyses of patients with RA. The independent associations of NT-proBNP levels and markers of disease activity with clinical cardiovascular endpoints need to be further investigated.

## Introduction

Patients with rheumatoid arthritis (RA) have a two- to three-fold increase in cardiovascular mortality and morbidity [1,2]. Several studies have reported an association between disease activity and cardiovascular mortality [3-5]. Population-based studies have also shown an increased prevalence of congestive heart failure (CHF) in patients with RA compared with healthy controls and patients with osteoarthritis [6,7]. Measures of disease activity and severity such as C-reactive protein (CRP), disability index, pain, and global severity are associated with an increased odds ratio for both concurrent and subsequent CHF [6,8].

The level of N-terminal pro-brain natriuretic peptide (NT-proBNP) predicts cardiac mortality and morbidity in the general population as well as in cohorts of patients with heart failure and stable coronary heart disease [9-12]. NT-proBNP, the biologically inactive N-terminal fragment of the active hormone BNP, has a longer half-life than the active hormone and is a viable biomarker of cardiovascular disease (CVD) [13]. In the healthy heart, the main site of BNP production is in the atrial cardiomyocytes, but as the heart fails fetal gene programs are activated and the ventricular myocardium becomes the main site of BNP production, releasing the peptide in response to stretch or ischemia [9,14].

The associations between clinical and laboratory markers of RA disease activity and levels of NT-proBNP have not been examined. There is also a lack of knowledge on the relationship between exposure to drugs used in the treatment of RA (glucocorticoids, disease-modifying anti-rheumatic drugs [DMARDs], non-steroidal anti-inflammatory drugs [NSAIDs], and cyclooxygenase-2 [COX-2] inhibitors) and NT-proBNP levels in patients with RA. Our objective, therefore, was to examine the cross-sectional and longitudinal associations between markers of inflammation, RA disease activity, medication used, and NT-proBNP levels (dependent variable).

## Materials and methods

### Patients

We followed 238 patients with RA of less than 4 years in duration longitudinally with comprehensive assessments of clinical and radiographic data at baseline and after 5 and 10 years [15]. The patients had all been recruited to the EUropean Research on Incapacitating DIsease and Social Support (EURIDISS) project, which was initially established in 1991. The project has been approved by the Norwegian Regional Committee for Research Ethics. All patients have signed an informed consent form on inclusion and at each follow-up assessment. At baseline, mean (range) age was 51.6 years (23 to 70), mean (standard deviation, SD) disease duration was 2.3 years (1.2), and 73.5% were female. Serum samples were collected from 237 patients at baseline, 126 patients after 5 years, and 145 after 10 years. Eighty-nine patients were lost to follow-up at the 10-year visit (42 declined to participate, 5 had moved out of the area, 35 had died, and 7 gave other reasons not to participate).

### Measures of disease activity

Disease activity was measured by joint tenderness, which was assessed at all examinations, and the Ritchie score was calculated [16]. Swollen joint counts were included at the 5- and 10-year assessments; that is, the disease activity score of 28 joints (DAS28) was not available from the baseline visit [17]. Surrogate measures of cumulative disease activity were health status and joint destruction. Health status was measured by the self-administered Stanford Health Assessment Questionnaire (HAQ) and the Arthritis Impact Measurement Scales 1 (AIMS1) [18,19]. Joint destruction was measured by radiographic damage of the hands and scored according to the van der Heijde modified Sharp criteria (referred to below as the Sharp score) [20]. Comorbidities were recorded by a trained study nurse at the 5- and 10-year follow-ups using a checklist modified from AIMS1 concerning the presence of 16 possible comorbidities. One of these items addresses the presence of angina, myocardial infarction, or other cardiac diseases. At the 10-year follow-up, known risk factors of CVD (hypertension, cholesterol, smoking, and creatinine) were also recorded. The use of NSAIDs, DMARDs, and glucocorticoids was recorded by the study nurse at all assessments (the use of COX-2 inhibitors was recorded only at the 10-year follow-up).

### Biomarkers

NT-proBNP was measured using a Modular E 170 device (Roche Diagnostics, Mannheim, Germany). The erythrocyte sedimentation rate was analyzed by the Westergren method at the time of examination, whereas the other biological markers were analyzed from frozen serum or plasma anti-cyclic citrullinated peptide antibody (anti-CCP) by enzyme-linked immunosorbent assay (ELISA) (Inova Diagnostics, San Diego, CA, USA) and CRP by phyCardioPhase high-sensitivity CRP nefelometri (Dade Behring, now part of Siemens AG, Munich, Germany). Rheumatoid factor (RF) IgM was analyzed using the ELISA method [21].

### Statistical analyses

NT-proBNP levels were dichotomized according to the national gender- and age-adjusted reference levels modified from Roche Diagnostics guidelines [22] (female: < 50 years of age, NT-proBNP ≤ 20 pmol/L; 50 to 70 years of age, ≤ 30 pmol/L; > 70 to 80 years of age, ≤ 100 pmol/L, and > 80 years of age, ≤ 200 pmol/L; male: < 50 years of age, NT-proBNP ≤ 10 pmol/L; 50 to 70 years of age, ≤ 20 pmol/L; > 70 to 80 years of age, ≤ 60 pmol/L, and > 80 years of age, ≤ 100 pmol/L). Groups were compared using the χ^2 ^and Mann-Whitney *U *tests as appropriate.

In the regression analyses, NT-proBNP was entered as a continuous dependent variable and was log-transformed to obtain normality. The presented β-coefficients have been transformed from the log value. All other variables were entered untransformed, and all analyses were adjusted for age and gender. The baseline cross-sectional associations between demographic, disease activity, and severity variables (gender, age, CRP, Sharp score of hands, Ritchie score, HAQ disease duration, RF IgM, anti-CCP, and use of DMARDs, steroids, or NSAIDs) and NT-proBNP levels were explored in bivariate linear regression analyses. Variables that were associated (*P *< 0.10) with NT-proBNP were then entered into a multivariate linear regression model and subsequently removed in a stepwise manner according to levels of significance. These analytic procedures were also applied to the cross-sectional data from the 10-year follow-up examination, which gave the opportunity of controlling not only for cardiovascular risk factors, including body mass index (BMI) and creatinine levels, but also for the presence of self-reported CVD.

Mixed model linear regression was used to examine longitudinal associations [23]. This is a longitudinal linear regression analysis that controls for multiple testing of the same patient by modelling the covariance between the repeated measurements of each individual as a clustered random effect. An unstructured covariance matrix was used in our analysis assuming that the correlation for each level of within-subject factors is different. The intra-subject variance is then used to calculate the standard error of the regression coefficient. The mixed model procedure deals with missing values by assuming them to be missing at random without removing the individual from the dataset. We investigated the change in NT-proBNP levels over time and examined associations between measures of inflammation, RA disease activity, medication taken, known CVD risk factors, and NT-proBNP (dependent variable). All analyses were performed using SPSS version 14 (SPSS Inc., Chicago, IL, USA). *P *values of less than 0.05 were considered significant.

## Results

At baseline, 35 patients had pathological levels of NT-proBNP. Disease characteristics were similar between these subgroups (normal versus elevated NT-proBNP), although the group with elevated NT-proBNP levels had significantly higher CRP levels (Table 1). CRP levels at baseline were significantly associated with NT-proBNP in the linear regression analyses (Table 2). This significant association was also maintained in the multivariate linear regression analyses, which included the interaction between age and gender as an effect modifier. The use of DMARDs, NSAIDs, or glucocorticoids was not significantly associated with NT-proBNP levels. The analysis was repeated with glucocorticoid use dichotomized according to a dosage greater than or less than 7.5 mg (*P *= 0.189).

**Table 1:** Baseline characteristics with comparison between patients with pathological versus normal levels of NT-proBNP

	All patients	NT-proBNP-negative^a ^n = 202	NT-proBNP-positive^a ^n = 35	*P *value
Age, years	51.9 (13.0)	51.4 (12.9)	54.3 (13.7)	0.17
Female gender	175 (73.5)	151 (74.8)	23 (65.7)	0.26
Disease duration, years	2.3 (1.2)	2.2 (1.1)	2.4 (1.3)	0.41
RF IgM-positive, ≥ 25	114 (47.9)	97 (48.0)	16 (45.7)	0.80
Anti-CCP-positive, ≥ 25	144 (60.5)	125 (61.9)	18 (51.4)	0.24
Sharp score	6.8 (11.8)	6.8 (12.3)	6.5 (8.1)	0.20
C-reactive protein, mg/L	9.9 (12.7)	9.2 (11.8)	13.9 (15.8)	0.05
DMARD user	124 (52.1)	104 (51.5)	19 (54.3)	0.76
NSAID user	109 (45.8	92 (45.5)	17 (48.6)	0.74
Glucocorticoid user, oral	65 (27.2)	51 (25.2)	14 (40)	0.07

Values are presented as mean (standard deviation) for continuous variables and as number (percentage) for counts. ^a^Age- and gender-adjusted reference levels. anti-CCP, anti-cyclic citrullinated peptide antibody; DMARD, disease-modifying anti-rheumatic drug; NSAID, non-steroidal anti-inflammatory drug; NT-proBNP, N-terminal pro-brain natriuretic peptide; RF, rheumatoid factor; Sharp score, van der Heijde modified Sharp criteria.

**Table 2:** Cross-sectional associations at baseline between NT-proBNP (dependent variable) and rheumatoid arthritis disease activity

	Adjusted linear regression		Multivariate linear regression	
	β coefficient	*P *value	β coefficient	*P *value

Age	1.02 (1.01–1.03)	< 0.001	1.009 (1.00–1.02)	0.04
Male gender	0.74 (0.59–0.95)	0.02	0.10 (0.04–0.23)	< 0.001
C-reactive protein^a^	1.01 (1.01–1.02)	0.002	1.01 (1.01–1.02)	0.001
RF IgM-positive^a, b^	0.96 (0.79–1.17)	0.67		
Anti-CCP-positive^a, b^	0.90 (0.74–1.10)	0.30		
Gluocorticoid user^a, b^	1.17 (0.94–1.48)	0.16		
DMARD user^a, b^	1.04 (0.85–1.26)	0.71		
NSAID user^a, b^	0.97 (0.80–1.19)	0.81		
Ritchie score^a^	1.01 (0.99–1.03)	0.21		
HAQ^a^	1.06 (0.90–1.24)	0.47		
Sharp score^a^	1.00 (0.99–1.02)	0.93		
Disease duration^a^	0.99 (0.91–1.08)	0.82		
Age × male gender			1.04 (1.02–1.05)	< 0.001

*R*^2 ^adjusted			0.23	< 0.05

^a^Age- and gender-adjusted; ^b^dichotomized variable. anti-CCP, anti-cyclic citrullinated peptide antibody; DMARD, disease-modifying anti-rheumatic drug; HAQ, Stanford Health Assessment Questionnaire; NSAID, non-steroidal anti-inflammatory drug; NT-proBNP, N-terminal pro-brain natriuretic peptide; RF, rheumatoid factor; Ritchie score, Ritchie articular index; Sharp score, van der Heijde modified Sharp criteria.

At the 10-year follow-up examination, age, gender, BMI, creatinine, CRP, and disease duration were significantly associated with NT-proBNP levels (Table 3). Consistent with our baseline analyses, CRP and NT-proBNP levels remained significantly associated in the multivariate analysis. The significant association between disease duration and NT-proBNP levels was also maintained. The model was also valid if self-reported CVD was entered as a covariate (β coefficient for CVD 1.65, 95% confidence interval [CI] 1.15 to 2.37; *P *= 0.007). The presence of hypertension, smoking status, and prevalent use of glucocorticoids, DMARDs (including subgroups of tumor necrosis factor [TNF] inhibitors and methotrexate users), COX-2 inhibitors, or NSAIDs was not significantly associated with NT-proBNP levels (data not shown).

**Table 3:** Cross-sectional associations at the 10-year follow-up between NT-proBNP (dependent variable) and rheumatoid arthritis disease activity, controlling for cardiovascular risk factors

	Adjusted linear regression		Multivariate linear regression	
	β coefficients	*P *value	β coefficients	*P *value

Age	1.04 (1.03–1.05)	< 0.001	1.04 (1.03–1.05)	< 0.001
Male gender	0.92 (0.64–1.31)	0.64	0.76 (0.53–1.04)	0.09
Body mass index^a^	0.96 (0.93–0.98)	0.001		
Atherogenic index^a^	0.95 (0.86–1.04)	0.25		
Creatinine^a^	1.02 (1.01–1.02)	0.001	1.01 (1.00–1.02)	0.04
C-reactive protein^a^	1.01 (1.0–1.03)	0.06	1.02 (1.00–1.02)	0.03
RF IgM-positive^a, b^	0.92 (0.72–1.19)	0.53		
Anti-CCP-positive^a, b^	1.14 (0.88–1.45)	0.33		
Ritchie score^a^	1.00 (0.99–1.01)	0.87		
DAS28^a^	1.05 (0.96–1.16)	0.27		
HAQ^a^	1.15 (0.94–1.41)	0.17		
Sharp score^a^	1.002 (1.00–1.01)	0.17		
Disease duration^a^	1.14 (1.02–1.27)	0.02	1.15 (1.03–1.27)	0.01

*R*^2 ^adjusted			0.38	< 0.001

^a^Age- and gender-adjusted; ^b^dichotomized variable. anti-CCP, anti-cyclic citrullinated peptide antibody; DAS28, disease activity score of 28 joints; HAQ, Stanford Health Assessment Questionnaire; NT-proBNP, N-terminal pro-brain natriuretic peptide; RF, rheumatoid factor; Ritchie score, Ritchie articular index; Sharp score, van der Heijde modified Sharp criteria.

In the repeated mixed model analysis, several markers of disease activity were associated with NT-proBNP levels, but after multivariate adjustment for time of follow-up, only CRP remained a significant predictor in the final model (Table 4). NT-proBNP levels increased between each follow-up visit and the interactions between age and gender and gender and time of follow-up were significant effect modifiers. The model remained significant if self-reported CVD at the 10-year follow-up was entered as a covariate (β coefficient for CVD 1.48, 95% CI 1.11 to 1.98; *P *= 0.008).

**Table 4:** Mixed model repeated measure linear regression showing the association between NT-proBNP (dependent variable) and markers of disease activity and treatment

	Adjusted linear regression (CI)		Multivariate linear regression (CI)	
	β coefficients (CI)	*P *value	β coefficients (CI)	*P *value

Age	1.03 (1.02–1.03)	< 0.001	1.02 (1.01–1.02)	< 0.001
Male gender	0.81 (0.64–1.01)	0.06	0.12 (0.05–0.26)	< 0.001
C-reactive protein^a^	1.01 (1.01–1.02)	< 0.001	1.01 (1.01–1.02)	< 0.001
HAQ^a^	1.12 (1.00–1.27)	0.06		
Prednisolone^a, b^	1.20 (1.03–1.41)	0.02		
NSAID^a, b^	0.90 (0.79–1.04)	0.14		
DMARD^a, b^	0.95 (0.83–1.09)	0.49		
Sharp score^a^	1.01 (1.00–1.01)	0.001		
Ritchie score^a^	1.01 (1.00–1.02)	0.01		
Time	1.04 (1.03–1.06)	< 0.001	1.03 (1.02–1.05)	< 0.001
Time × male gender			1.04 (1.01–1.07)	0.01
Age × male gender			1.03 (1.02–1.05)	0.01

^a^Age- and gender-adjusted; ^b^dichotomized variable. CI, confidence interval; DMARD, disease-modifying anti-rheumatic drug; HAQ, Stanford Health Assessment Questionnaire; NSAID, non-steroidal anti-inflammatory drug; NT-proBNP, N-terminal pro-brain natriuretic peptide; Ritchie score, Ritchie articular index; Sharp score, van der Heijde modified Sharp criteria.

## Discussion

NT-proBNP has been shown to be an independent predictor of cardiovascular morbidity and mortality [9-11], and disease activity in RA correlates with mortality, CVD, and CHF [5,6]. Hence, it is of interest to investigate how markers of inflammation and disease activity are related to NT-proBNP in a longitudinal perspective. We found significant cross-sectional and longitudinal associations between CRP and levels of NT-proBNP in this cohort of RA patients with less than 4 years of disease duration at inclusion. CRP remained an independent predictor of NT-proBNP levels after controlling for known cardiovascular risk factors and the presence of self-reported CVD at the 10-year follow-up and in the longitudinal analyses.

Our findings are intriguing as the relationship between BNP/NT-proBNP values and the risk of CVD may not be dependent on a threshold value of BNP/NT-proBNP but rather represents a continuum [11,24]. Wang and colleagues [11] found that a single measurement of BNP provided prognostic information in an unselected population of 3,346 persons without heart failure followed for a mean of 5.2 years. An increase of BNP levels by 1 SD was associated with a 77% increased risk of developing heart failure and a 28% increased risk of a first cardiovascular event [11]. BNP has been validated as a marker of CVD in patients with RA. Harney and colleagues [25] found that BNP levels were significantly higher in 26 patients with RA than in controls and correlated with end diastolic volume (*r*^2 ^= 0.83), end systolic volume (*r*^2 ^= 0.62), and left ventricular mass (*r*^2 ^= 0.4). Levels of NT-proBNP have been found to correlate closely with BNP (*r *= 0.9; *P *< 0.001) [24].

Increased levels of inflammatory markers are associated with an increased risk of cardiovascular morbidity and mortality [26,27]. We found that CRP was significantly associated with levels of NT-proBNP at all time points. Flares in inflammatory activity might be better predictors of cardiovascular events than long-term exposure to low-grade inflammation. Indeed, there is evidence that the initiation of CHF in RA patients is preceded by a flare in disease activity [8]. Radiographic progression and the HAQ score are surrogate measures of cumulative disease activity, although we acknowledge that there are wide inter-individual variations [28-31]. We found no significant association between these measures and NT-proBNP levels in our multivariate longitudinal analyses. Previous reports on the association between joint destruction and CVD in RA have been inconsistent; Wållberg-Jonsson and colleagues [5] demonstrated that early progression of erosions increased the risk of cardiovascular events. Solomon and colleagues [1], on the other hand, did not find that joint erosions were significantly associated with cardiovascular death or myocardial infarction in multivariate analyses. However, we did find disease duration to be significantly associated with NT-proBNP levels at the 10-year cross-sectional analysis. As all patients had a disease duration of 4 years or less at inclusion, the correlation with NT-proBNP levels after 10 years is difficult to interpret. A link between RA disease duration and increased CVD risk can be questioned. In the Rochester cohort, no association was found between risk of heart failure or cardiovascular death and RA disease duration [7,32], nor did Book and colleagues [33] find an association between disease duration and mortality.

To our knowledge, no previous study has examined the association between markers of inflammation in RA and BNP/NT-proBNP. It could be argued that the association between CRP and NT-proBNP identified in our study is a consequence of the inflammatory nature of ventricular remodelling. Patients with RA are not only exposed to systemic inflammation from their arthritic disease, the failing heart itself may be invaded by peripheral blood mononuclear cells that participate in left ventricular remodelling. These and other inflammatory cells release TNF-α and interleukin-6, thereby increasing CRP levels through upregulation of hepatic synthesis [27].

The strength of the present study is the prospective longitudinal design with regular clinical examinations, blood sampling with subsequent batch analyses, and documentation of radiographic progression. NT-proBNP has been shown to tolerate routine laboratory handling and may be analyzed from frozen sera [10,34]. The validity of our model is supported by the association between NT-proBNP levels and self-reported CVD. Furthermore, in agreement with previous studies, creatinine levels predict NT-proBNP in the final model. NT-proBNP is metabolized in the renal proximal tubule, and reduced renal function partially accounts for the age-related increase in NT-proBNP levels in the general population [14,35]. We found BMI to be inversely correlated with NT-proBNP levels in the adjusted bivariate analyses although it did not reach significance in the multivariate analysis. Other studies have confirmed the inverse relationship between BMI and NT-proBNP levels [36].

This study has several shortcomings. Two hundred thirty-eight patients were included at baseline, and 145 completed the 10-year follow-up. The small scale of this study is a limitation in the analyses. CVD in RA was not a major focus when the cohort was established, and information relating to cardiovascular comorbidity, smoking, concomitant medication, blood pressure, or BMI was not recorded at baseline. The presence of CVD was recorded by a questionnaire at the 10-year follow-up and we did not have the opportunity to verify the diagnosis by searching the medical records. Cause of death for the 35 patients who died during the study was not recorded. Thus, we considered that the data were not sufficiently robust to allow an explorative analysis of how levels of NT-proBNP predict future CVD. However, the patients were requested to report current medication. There was perfect agreement between self-reported CVD and the reported prescription of medication typically used to treat CVD, such as anti-hypertensive drugs, anti-platelet agents, or nitrates, allowing for an internal consistency check.

## Conclusion

CRP levels were linearly associated with levels of NT-proBNP in this cross-sectional and longitudinal study of 238 patients with RA. Further studies are needed to elucidate how this biomarker can be implemented in clinical decision making in RA.

## Abbreviations

AIMS1 = Arthritis Impact Measurement Scales 1; anti-CCP = anti-cyclic citrullinated peptide antibody; BMI = body mass index; BNP = brain natriuretic peptide; CHF = congestive heart failure; CI = confidence interval; COX-2 = cyclooxygenase-2; CRP = C-reactive protein; CVD = cardiovascular disease; DAS28 = disease activity score of 28 joints; DMARD = disease-modifying anti-rheumatic drug; ELISA = enzyme-linked immunosorbent assay; HAQ = Stanford Health Assessment Questionnaire; NSAID = non-steroidal anti-inflammatory drug; NT-proBNP = N-terminal pro-brain natriuretic peptide; RA = rheumatoid arthritis; RF = rheumatoid factor; SD = standard deviation; Sharp score = van der Heijde modified Sharp criteria; TNF = tumor necrosis factor.

## Competing interests

The authors declare that they have no competing interests.

## Authors' contributions

SAP developed the study, performed the analyses, and drafted the manuscript. KA participated in the development of the study and in the drafting of the manuscript. SØ participated in the data collection and the drafting of the manuscript. PM gave statistical advice and helped in the drafting of the manuscript. DA conceived the study, participated in its design and development, and helped in the drafting of the manuscript. TKK conceived the study, participated in its design and development, and helped in the drafting of the manuscript. All authors have read and approved the manuscript.

## References

[B1] Solomon DH, Karlson EW, Rimm EB, Cannuscio CC, Mandl LA, Manson JE, Stampfer MJ, Curhan GC (2003). Cardiovascular morbidity and mortality in women diagnosed with rheumatoid arthritis. Circulation.

[B2] del Rincón ID, Williams K, Stern MP, Freeman GL, Escalante A (2001). High incidence of cardiovascular events in a rheumatoid arthritis cohort not explained by traditional cardiac risk factors. Arthritis Rheum.

[B3] Turesson C, McClelland RL, Christianson TJ, Matteson EL (2007). Severe extra-articular disease manifestations are associated with an increased risk of first ever cardiovascular events in patients with rheumatoid arthritis. Ann Rheum Dis.

[B4] Farragher TM, Lunt M, Bunn DK, Silman AJ, Symmons DP (2007). Early functional disability predicts both all-cause and cardiovascular mortality in people with inflammatory polyarthritis: results from the Norfolk Arthritis Register. Ann Rheum Dis.

[B5] Wållberg-Jonsson S, Johansson H, Ohman ML, Rantapää-Dahlqvist S (1999). Extent of inflammation predicts cardiovascular disease and overall mortality in seropositive rheumatoid arthritis. A retrospective cohort study from disease onset. J Rheumatol.

[B6] Wolfe F, Michaud K (2004). Heart failure in rheumatoid arthritis: rates, predictors, and the effect of anti-tumor necrosis factor therapy. Am J Med.

[B7] Nicola PJ, Maradit-Kremers H, Roger VL, Jacobsen SJ, Crowson CS, Ballman KV, Gabriel SE (2005). The risk of congestive heart failure in rheumatoid arthritis: a population-based study over 46 years. Arthritis Rheum.

[B8] Maradit-Kremers H, Nicola PJ, Crowson CS, Ballman KV, Jacobsen SJ, Roger VL, Gabriel SE (2007). Raised erythrocyte sedimentation rate signals heart failure in patients with rheumatoid arthritis. Ann Rheum Dis.

[B9] Bibbins-Domingo K, Ansari M, Schiller NB, Massie B, Whooley MA (2003). B-type natriuretic peptide and ischemia in patients with stable coronary disease: data from the Heart and Soul study. Circulation.

[B10] Kragelund C, Gronning B, Kober L, Hildebrandt P, Steffensen R (2005). N-terminal pro-B-type natriuretic peptide and long-term mortality in stable coronary heart disease. N Engl J Med.

[B11] Wang TJ, Larson MG, Levy D, Benjamin EJ, Leip EP, Omland T, Wolf PA, Vasan RS (2004). Plasma natriuretic peptide levels and the risk of cardiovascular events and death. N Engl J Med.

[B12] Bettencourt P, Azevedo A, Pimenta J, Frioes F, Ferreira S, Ferreira A (2004). N-terminal-pro-brain natriuretic peptide predicts outcome after hospital discharge in heart failure patients. Circulation.

[B13] Tschope C, Kasner M, Westermann D, Gaub R, Poller WC, Schultheiss HP (2005). The role of NT-proBNP in the diagnostics of isolated diastolic dysfunction: correlation with echocardiographic and invasive measurements. Eur Heart J.

[B14] McKie PM, Burnett JC (2005). B-type natriuretic peptide as a biomarker beyond heart failure: speculations and opportunities. Mayo Clin Proc.

[B15] Smedstad LM, Vaglum P, Kvien TK, Moum T (1995). The relationship between self-reported pain and sociodemographic variables, anxiety, and depressive symptoms in rheumatoid arthritis. J Rheumatol.

[B16] Ritchie DM, Boyle JA, McInnes JM, Jasani MK, Dalakos TG, Grieveson P, Buchanan WW (1968). Clinical studies with an articular index for the assessment of joint tenderness in patients with rheumatoid arthritis. Q J Med.

[B17] Prevoo ML, van 't Hof MA, Kuper HH, van Leeuwen MA, Putte LB van de, van Riel PL (1995). Modified disease activity scores that include twenty-eight-joint counts. Development and validation in a prospective longitudinal study of patients with rheumatoid arthritis. Arthritis Rheum.

[B18] Fries JF, Spitz P, Kraines RG, Holman HR (1980). Measurement of patient outcome in arthritis. Arthritis Rheum.

[B19] Meenan RF, Gertman PM, Mason JH (1980). Measuring health status in arthritis. The arthritis impact measurement scales. Arthritis Rheum.

[B20] Heijde DM van der (1996). Plain X-rays in rheumatoid arthritis: overview of scoring methods, their reliability and applicability. Baillieres Clin Rheumatol.

[B21] Syversen SW, Gaarder PI, Goll GL, Ødegård S, Haavardsholm EA, Mowinckel P, Heijde D van der, Landewé R, Kvien TK (2008). High anti-cyclic citrullinated peptide levels and an algorithm of four variables predict radiographic progression in patients with rheumatoid arthritis: results from a 10-year longitudinal study. Ann Rheum Dis.

[B22] Raymond I, Groenning BA, Hildebrandt PR, Nilsson JC, Baumann M, Trawinski J, Pedersen F (2003). The influence of age, sex and other variables on the plasma level of N-terminal pro brain natriuretic peptide in a large sample of the general population. Heart.

[B23] Chan YH (2004). Biostatistics 301A. Repeated measurement analysis (mixed models). Singapore Med J.

[B24] Richards M, Nicholls MG, Espiner EA, Lainchbury JG, Troughton RW, Elliott J, Frampton CM, Crozier IG, Yandle TG, Doughty R, MacMahon S, Sharpe N, Christchurch Cardioendocrine Research Group; Australia-New Zealand Heart Failure Group (2006). Comparison of B-type natriuretic peptides for assessment of cardiac function and prognosis in stable ischemic heart disease. J Am Coll Cardiol.

[B25] Harney SM, Timperley J, Daly C, Harin A, James T, Brown MA, Banning AP, Fox K, Donnelly S, Wordsworth BP (2006). Brain natriuretic peptide is a potentially useful screening tool for the detection of cardiovascular disease in patients with rheumatoid arthritis. Ann Rheum Dis.

[B26] Ridker PM, Buring JE, Cook NR, Rifai N (2003). C-reactive protein, the metabolic syndrome, and risk of incident cardiovascular events: an 8-year follow-up of 14 719 initially healthy American women. Circulation.

[B27] Vasan RS, Sullivan LM, Roubenoff R, Dinarello CA, Harris T, Benjamin EJ, Sawyer DB, Levy D, Wilson PW, D'Agostino RB, Framingham Heart Study (2003). Inflammatory markers and risk of heart failure in elderly subjects without prior myocardial infarction: the Framingham Heart Study. Circulation.

[B28] van Leeuwen MA, van Rijswijk MH, Heijde DM van der, Te Meerman GJ, van Riel PL, Houtman PM, Putte LB van De, Limburg PC (1993). The acute-phase response in relation to radiographic progression in early rheumatoid arthritis: a prospective study during the first three years of the disease. Br J Rheumatol.

[B29] Wick MC, Lindblad S, Klareskog L, Van Vollenhoven RF (2004). Relationship between inflammation and joint destruction in early rheumatoid arthritis: a mathematical description. Ann Rheum Dis.

[B30] van Leeuwen MA, van Rijswijk MH, Sluiter WJ, van Riel PL, Kuper IH, Putte LB van de, Pepys MB, Limburg PC (1997). Individual relationship between progression of radiological damage and the acute phase response in early rheumatoid arthritis. Towards development of a decision support system. J Rheumatol.

[B31] Ødegård S, Landewé R, Heijde  van der, Kvien TK, Mowinckel P, Uhlig T (2006). Association of early radiographic damage with impaired physical function in rheumatoid arthritis: a ten-year, longitudinal observational study in 238 patients. Arthritis Rheum.

[B32] Maradit-Kremers H, Nicola PJ, Crowson CS, Ballman KV, Gabriel SE (2005). Cardiovascular death in rheumatoid arthritis: a population-based study. Arthritis Rheum.

[B33] Book C, Saxne T, Jacobsson LT (2005). Prediction of mortality in rheumatoid arthritis based on disease activity markers. J Rheumatol.

[B34] Hobbs FD, Davis RC, Roalfe AK, Hare R, Davies MK, Kenkre JE (2002). Reliability of N-terminal pro-brain natriuretic peptide assay in diagnosis of heart failure: cohort study in representative and high risk community populations. BMJ.

[B35] Galasko GI, Lahiri A, Barnes SC, Collinson P, Senior R (2005). What is the normal range for N-terminal pro-brain natriuretic peptide? How well does this normal range screen for cardiovascular disease?. Eur Heart J.

[B36] St Peter JV, Hartley GG, Murakami MM, Apple FS (2006). B-type natriuretic peptide (BNP) and N-terminal pro-BNP in obese patients without heart failure: relationship to body mass index and gastric bypass surgery. Clin Chem.

